# Melatonin MT_1_ and MT_2_ Receptors in the Ram Reproductive Tract

**DOI:** 10.3390/ijms18030662

**Published:** 2017-03-19

**Authors:** Marta González-Arto, David Aguilar, Elena Gaspar-Torrubia, Margarita Gallego, Melissa Carvajal-Serna, Luis V. Herrera-Marcos, Edith Serrano-Blesa, Thais Rose dos Santos Hamilton, Rosaura Pérez-Pé, Teresa Muiño-Blanco, José A. Cebrián-Pérez, Adriana Casao

**Affiliations:** 1 Grupo Biología y Fisiología de la Reproducción, Instituto de Investigación de Ciencias Ambientales de Aragón (IUCA), Facultad de Veterinaria, Universidad de Zaragoza, 50013 Zaragoza, Spain; martagonzalezarto@hotmail.com (M.G.-A.); d.aguilar27@hotmail.com (D.A.); anaelenagaspar@gmail.com (E.G.-T.); e.serranoblesa@gmail.com (E.S.-B.); rosperez@unizar.es (R.P.-P.); muino@unizar.es (T.M.-B.); pcebrian@unizar.es (J.C.-P.); 2 Departamento de Patología Animal, Facultad de Veterinaria, Universidad de Zaragoza, 50013 Zaragoza, Spain; mgallego@unizar.es; 3 Departamento de Producción Animal, Facultad de Medicina Veterinaria y de Zootecnia, Universidad Nacional de Colombia, 11001 Bogotá, Colombia; mcarvajals@unal.edu.co; 4 Departamento de Bioquímica y Biología Molecular y Celular, Facultad de Veterinaria, Instituto de Investigación Sanitaria de Aragón-Universidad de Zaragoza, 50013 Zaragoza, Spain; l.vte.herrera@gmail.com; 5 Dpto. de Reprodução Animal, da Faculdade de Medicina Veterinaria e Zootecnia, da Universidade de São Paulo, 05508 270 São Paulo, Brazil; thaisroseh@gmail.com

**Keywords:** melatonin receptor, ram, testis, epididymis, accessory glands

## Abstract

Some melatonin functions in mammals are exerted through MT_1_ and MT_2_ receptors. However, there are no reports of their presence in the reproductive tract of the ram, a seasonal species. Thus, we have investigated their existence in the ram testis, epididymis, accessory glands and ductus deferens. Real-time polymerase chain reaction (qPCR) revealed higher levels of m-RNA for both receptors in the testis, ampulla, seminal vesicles, and vas deferens, than in the other organs of the reproductive tract (*p* < 0.05). Western blot analyses showed protein bands compatible with the MT_1_ in the testis and cauda epididymis, and for the MT_2_ in the cauda epididymis and deferent duct. Immunohistochemistry analyses revealed the presence of MT_1_ receptors in spermatogonias, spermatocytes, and spermatids, and MT_2_ receptors in the newly-formed spermatozoa in the testis, whereas both receptors were located in the epithelial cells of the ampulla, seminal vesicles, and ductus deferens. Indirect immunofluorescence showed significant differences in the immunolocation of both receptors in spermatozoa during their transit in the epididymis. In conclusion, it was demonstrated that melatonin receptors are present in the ram reproductive tract. These results open the way for new studies on the molecular mechanism of melatonin and the biological significance of its receptors.

## 1. Introduction

Melatonin is a multifunctional molecule, widely distributed among all taxa [1]. In unicellular organisms its main function seems to be protection against free radical damage [2], while in mammals it has an extensive range of functions, including the regulation of circadian rhythms, immunomodulation, cancer inhibition, gastrointestinal tract protection, cardiovascular regulation, and seasonal reproduction [3].

Melatonin can exert many of these functions due to its antioxidant properties [4], but it can also interact with cytosolic molecules, such as calmodulin [5] or tubulin [6]. Likewise, melatonin can also act through its union with the membrane receptors MT_1_ and MT_2_ [7], and the MT_3_/QR2 [8]. Melatonin receptors are located in the suprachiasmatic nucleus to regulate the circadian pacemaker but are also present in peripheral tissues, such as blood vessels for vasomotor control, or white and bone marrow cells for regulation of the immune system [9].

Melatonin receptors have also been identified in the reproductive system. Until recently, MT_1_ and/or MT_2_ have been located in the cumulus-oocyte complexes [10], granulosa and luteal cells of the ovary in humans [11] and in rats [12], in the human myometrium [13], and breast epithelial cells [14], as well as in the placenta [15]. In the male, melatonin receptors have been identified in rat [16] and hamster [17] testis by reverse transcription polymerase chain reaction (RT-PCR) and immunohistochemistry techniques, in the rat epididymis by means of melatonin receptor agonists [18], by 2-[^125^I] iodomelatonin, and in human prostate epithelial cells [19] and in the prostatic section of the ductus deferens [20]. However, there is no evidence of the presence of melatonin receptors in the other accessory glands of the male reproductive tract (ampulla, seminal vesicle, or bulbourethral glands). Moreover, there are no reports of the presence of melatonin receptors in the ram reproductive tract despite the great importance of melatonin in sheep reproduction.

The ovine is a seasonal species, in which seasonality is regulated by the nocturnal secretion of melatonin by the pineal gland [21]. This increase in plasma melatonin levels during the autumn/winter nights will stimulate the hypothalamus-pituitary-gonadal axis [22], marking the beginning of the reproductive season. Although seasonality is less marked in the ram than in the ewe [21], there is a decrease in the testicular volume and sexual behavior during the non-reproductive season [23] in the male, along with a decrease in sperm quality and functionality [24]. This decline of the ram reproductive parameters has been previously related with a concomitant decrease in the activity of the hypothalamus-pituitary-testicular axis and blood testosterone levels [25].

However, recent studies suggest more direct action of melatonin on the spermatogenesis and ram sperm functionality. Thus, exogenous melatonin treatment during the non-reproductive season can increase both the ram ejaculate volume and total spermatozoa number [26], along with sperm motility parameters [27] and concentration [28]. Although all of these positive effects exerted by melatonin treatment in the ram could be explained by the increase in testosterone levels provoked by this hormone [29], the possibility of direct receptor-mediated melatonin action on the ram testes cannot be ruled out.

Moreover, we have previously identified the presence of both melatonin receptors MT_1_ and MT_2_ in ram spermatozoa [30] and proved that melatonin can inhibit apoptosis [31] and modulate ram sperm capacitation through the MT_2_ receptor [32]. We have also measured seasonal variations of the melatonin concentration in ram seminal plasma [33], and we have recently demonstrated that the melatonin synthesizing enzymes are expressed in the ram testes [34], mainly in the Leydig cells, spermatocytes and spermatids. The testicular melatonin could protect the developing spermatozoa from oxidative damage [35], one of the possible mechanisms of action being through melatonin receptors in the cells [36].

Additionally, there are extensive reports of seasonal variations in the composition of ram seminal plasma [37], and of how melatonin treatment modifies its biochemical composition [38]. In fact, melatonin treatment reactivates the seminal vesicles during the non-reproductive season [39], increasing their size [40]. It also modulates the contraction of the vas deferens [41]. Therefore, we can hypothesize that melatonin could act directly on the accessory glands, possibly by melatonin receptor binding.

Given the lack of information on the presence of melatonin receptors in the ram reproductive tract, and for further elucidation of the molecular mechanism of melatonin in the male of this species, the objective of this work is to determine the presence of MT_1_ and MT_2_ receptors in the ram testis, epididymis, and accessory sex glands.

## 2. Results

### 2.1. Melatonin Receptor Gene Expression in the Ram Reproductive Tract

The real-time polymerase chain reaction (qPCR) analysis detected the transcripts for both melatonin receptors in all the studied tissues, but also revealed substantial differences between them in gene expression.

**Figure 1:**
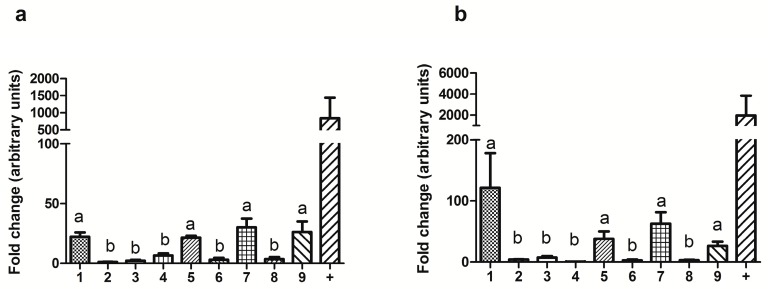
Real-time polymerase chain reaction (qPCR) analysis of (**a**) MT_1_ and (**b**) MT_2_ melatonin receptors from mRNA obtained from various tissues of the ram reproductive tract: Testis (1), caput (2), corpus (3) and cauda epididymis (4), ampulla (5), prostate (6), seminal vesicles (7), bulbourethral glands (8), and ductus deferens (9) . Ovine granulosa cells and ovine retina were used as positive control (+) for (**a**) MT_1_ and (**b**) MT_2_, respectively. Values of mRNA relative expression are shown as mean ± SEM of two males analyzed twice (*n* = 4). Each qPCR run was performed in triplicate. Glyceraldehyde-3-phosphate dehydrogenase (*GAPDH*) and *β-actine* genes were used as housekeeping genes. Different letters a, b mean statistical differences of *p* < 0.05 (positive controls were only used for representative purposes, and were not included in the statistical analyses).

The testis, ampulla, seminal vesicle and vas deferens showed significantly higher (*p* < 0.05) gene expression levels of MT_1_ and MT_2_ than those in the other tissues of the male genital tract (Figure 1).

### 2.2. Protein Expression by Western-Blot and Immunohistochemistry

**Figure 2:**
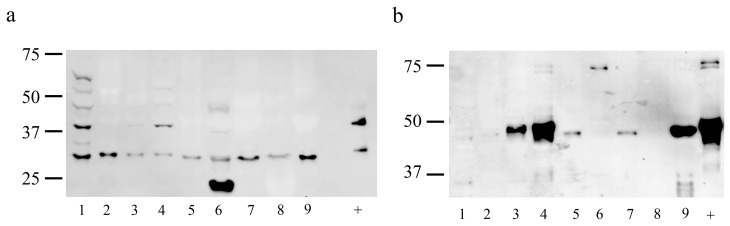
Western blot analysis of (**a**) MT_1_ and (**b**) MT_2_ in proteins obtained from various tissues of the ram reproductive tract: Testis (1), caput (2), corpus (3) and cauda epididymis (4), ampulla (5), prostate (6), seminal vesicles (7), bulbourethral glands (8), ductus deferens (9), and spermatozoa (+, positive control).

Western blot analyses of the proteins extracted from ram reproductive tissues revealed a strong 39 kDa band, compatible with the MT_1_ melatonin receptor, in the testis and cauda epididymis, and a fainter one in the corpus and the prostate (Figure 2a). A smaller band of 32 kDa was also found in all of the studied tissues. Both the 39 and 32 kDa signals were also found in the spermatozoa (positive control), along with a very faded band of 50 kDa, which was also present in the testis, epididymis, and prostate. Likewise, several weaker bands of molecular weight ranging from 35 to 60 kDa were found, mainly in the testis, but also to a lesser extent in the cauda epididymis and in the prostate. This organ also showed a very strong band of 23 kDa.

Western blot analyses against the MT_2_ melatonin receptor revealed a very faint 39 kDa protein band, compatible with this receptor, in the cauda epididymis, deferent duct, and spermatozoa (positive control, Figure 2b). The positive control also showed a very strong signal that was composed of a double band of 45–50 kDa, as we previously described [30]. A similar band of 45–50 kDa was found in the cauda, and a smaller one of 48 kDa in the corpus. A strong band of 46 kDa was detected in the deferent duct, along with narrower bands of the same molecular weight in the ampulla and the seminal vesicles, and very faint ones in the testis and the caput epididymis. In the testis and the deferent duct, a smaller band of 35 kDa was identified. Finally, a double 75 kDa band was found in the cauda epididymis and the positive control, and a single one in the prostate.

**Figure 3:**
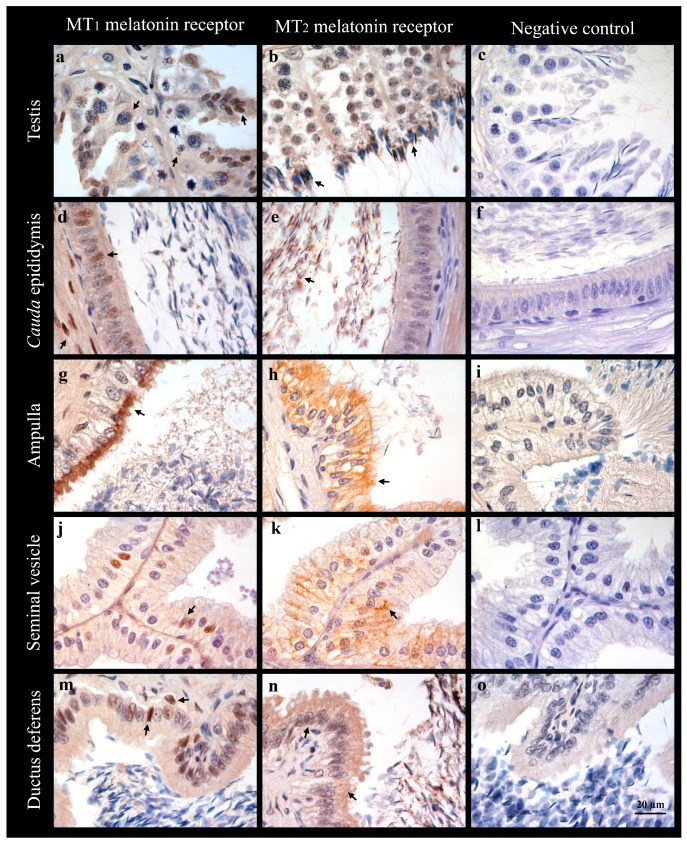
Immunohistochemical localization using the avidin-biotin complex technique of MT_1_ (arrows in panels **a**, **d**, **g**, **j** and **m**) and MT_2_ (arrows in panels **b**, **e**, **h**, **k**, and **n**) in testis (**a**–**c**), cauda epididymis (**d**–**f**), ampulla (**g**–**i**), seminal vesicles (**j**–**l**), and ductus deferens (**m**–**o**). Magnification 1000×. A 20 µm measurement bar is displayed in panel **o**. Negative controls are shown (panels **c**, **f**, **i**, **l**, and **o**).

In order to corroborate the data found with qPCR and Western-blot analyses, we performed an immunohistochemistry study using the avidin-biotin complex technique on the tissues with the highest gene and/or protein expression of the melatonin receptors, namely the testis, cauda epididymis, ampulla, seminal vesicles, and vas deferens (Figure 3).

In the testis, positivity for MT_1_ was found in the form of dark brown spots in the cytoplasm located around the nucleus of the spermatogonias and spermatocyte. Likewise, round and maturing spermatids showed an intense immunostaining in the nucleus and cytoplasm (Figure 3a). No immunostaining for MT_1_ or MT_2_ was detected in the Sertoli or Leydig cells. A clear immunoreactivity for MT_2_ was only found in the neck and head of the newly-formed spermatozoa (Figure 3b).

In the cauda epididymis, the MT_1_ receptor was found as a brown stain in the nucleus of some of the columnar epithelial cells (Figure 3d). This positivity was also found in the nucleus of the smooth muscle cells (positive control). On the other hand, for the MT_2_ receptor, only the spermatozoa contained in the cauda epididymis lumen showed some positivity (Figure 3e).

In the ampulla, positivity for MT_1_ was found in the apical pole of the epithelial cells (Figure 3g). However, a positive reaction for MT_2_ was detected within all the cytoplasm of the cells (Figure 3h).

Similarly, the MT_1_ receptor was found in the nucleus of a few cells on the pseudostratified columnar epithelium of the seminal vesicles (Figure 3i), whereas the MT_2_ receptor was located in the cytoplasm of these cells (Figure 3j). It is worth noting that positive and negative secretory alveoli coexisted. In addition, alveoli, which showed both positive and negative secretory cells, were also observed.

In the ductus deferens, immunostaining for the MT_1_ receptor was found in the nucleus of the pseudostratified columnar epithelium and basal cells (Figure 3l). Although nearly all of these cells seem to be positive to the MT_1_ receptor, differences in staining intensity can also be seen. Smooth muscle cells are also positive for the MT_1_ receptor (positive control). In this tissue, positivity for the MT_2_ receptor is located in the cytoplasm of the epithelial cells, between the nucleus and the apical pole. The nucleus of the cells is also stained to a lesser extent (Figure 3m).

### 2.3. Changes in Melatonin Receptor Distribution during Ram Sperm Maturation in the Testis and Epididymis

Despite the low gene expression for MT_1_ and MT_2_ receptors found in the epididymis, we observed high protein expression of these receptors in this organ by means of Western blot. Furthermore, we also detected differences in the Western blot band pattern between the testis, caput, corpus, and cauda epididymis. The immunohistochemistry (IHC) analyses, especially against the MT_2_ receptor, suggested that these differences might be due to the spermatozoa contained in the lumen of these organs. Consequently, we decided to determine whether melatonin receptor expression or distribution varies during ram sperm maturation.

For the melatonin receptor distribution analysis, spermatozoa were classified according to a previous study performed on ejaculated spermatozoa [30]: In the case of the MT_1_ receptor, spermatozoa were categorized in four immunotypes; Type I labelled all over the head and tail, type II with reactivity at the equatorial and post-acrosomal regions, neck and tail; type III labelled only at the equatorial zone and tail, and type IV stained only on the tail. Indirect immunofluorescence (IIF) against the MT_2_ melatonin receptor allowed us to classify the spermatozoa into four different immunotypes: Type A, showing greater intensity of staining at the acrosome than the post-acrosome; type P, with more intense staining at the post-acrosome region than the acrosome; type AP, with the same intensity of immunostaining at both acrosome and post-acrosome, and a fourth immunotype, not described in ejaculated spermatozoa, named type N, with staining only in the neck. Additionally, spermatozoa without immunoreactivity were recorded for both receptors. According to this sorting, 27.3% ± 3.5% and 43.0% ± 3.6% of the spermatozoa obtained from the testis showed no immunostaining against the MT_1_ and MT_2_ receptors, respectively, whereas in the caput epididymis this percentage decreased to 7.3% ± 5.3% and 3.0% ± 3.0%, respectively (*p* < 0.001). In the corpus, and cauda epididymis, all the cells were positive to the presence of the receptors.

**Figure 4:**
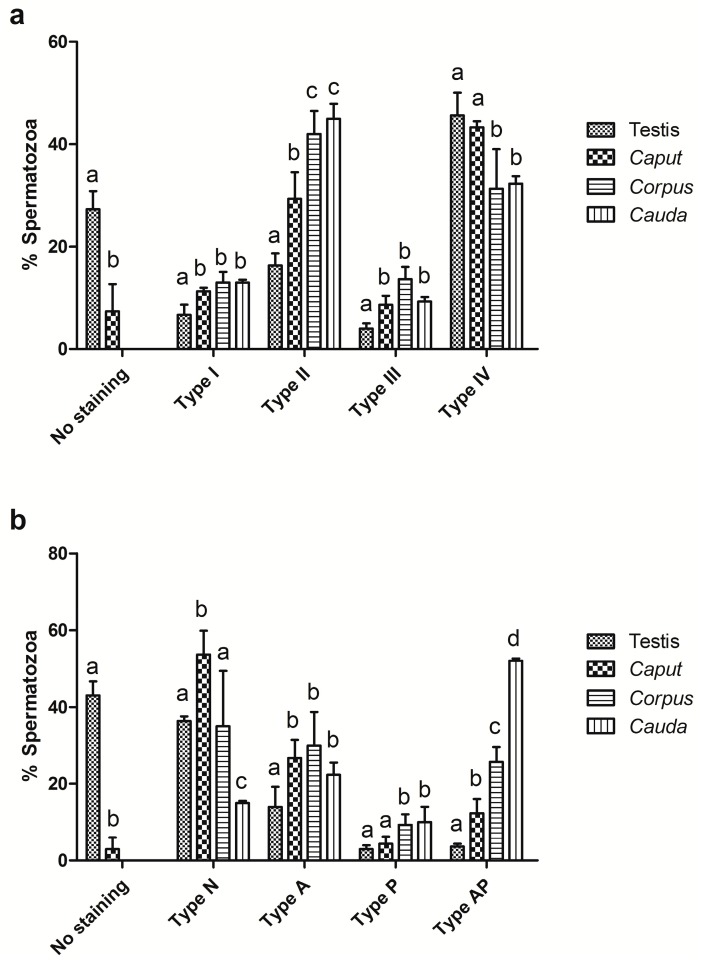
Percentages of the different immunotypes, assessed by indirect immunofluorescence (IIF) against MT_1_ (**a**) and MT_2_ (**b**) melatonin receptors in spermatozoa obtained from the testis and the caput, corpus, and cauda epididymis. For the MT_1_ receptor, type I (labelling all over the head and tail), type II (equatorial and post-acrosomal regions, neck, and tail), type III (equatorial zone and tail), and type IV (staining only on the tail) were detected. For the MT_2_ melatonin receptor, type A (more intense staining at the acrosome than the post-acrosome), type P (more intense staining at the post-acrosome region than the acrosome), type AP (the same intensity of immunostaining at both acrosome and post-acrosome), and type N (staining only in the neck) spermatozoa were identified. Additionally, spermatozoa without immunoreactivity were recorded for both receptors. Results are shown as mean ± SEM, *n* = 5. Different letters (a, b, c) mean statistical differences of *p* < 0.05.

In the testis, the predominant immunotype regarding the MT_1_ receptor distribution was type IV (45.6% ± 4.4%). This immunotype decreased during the sperm maturation in the epididymis (43.3% ± 1.2%, 31.3% ± 7.7%, and 32.5% ± 1.4% for the caput, corpus, and cauda epididymis, respectively; *p* < 0.05 when corpus and cauda are compared with the testis and caput). Concomitantly, the percentage of type II increased (16.3% ± 2.3%, 29.3% ± 5.2%, 42.0% ± 4.5%, and 45.0% ± 2.8% for the testis, caput, corpus, and cauda epididymis, respectively; *p* < 0.001 when the epididymis is compared with the testis, and *p* < 0.01 when the caput epididymis is compared with the corpus and *cauda*, respectively). There were no significant differences in the percentage of type I (6.6% ± 2.0%, 11.3% ± 0.6%, 13.0% ± 2.0%, and 13.1% ± 0.5% for testis, caput, corpus, and cauda epididymis) or type III spermatozoa (4.0% ± 1.0%, 8.6% ± 1.7%, 13.6% ± 2.4%, and 9.3% ± 0.8% for testis, caput, corpus, and cauda epididymis) during sperm maturation in the epididymis, but their percentages showed significant differences when compared with the testis (*p* < 0.05, Figure 4a).

Similarly, the analysis of the MT_2_ receptor distribution revealed that the predominant immunotype in the testis was type N (36.3% ± 1.2%, Figure 4b).This immunotype was increased in the caput epididymis (53.6% ± 6.1%; *p* < 0.001, when compared with the testis), mainly due to the high decrease in the percentage of non-stained spermatozoa, and decreased during sperm maturation (35.0% ± 14.3% and 15.0% ± 0.5% for the corpus and cauda epididymis, respectively; *p* < 0.001 for the corpus when compared with the cauda, and the cauda when compared with the testis and caput epididymis, respectively). Alongside this type N decrease, type AP increased (3.6% ± 0.6%, 12.3% ± 3.7%, 25.6% ± 3.9%, and 52.5% ± 0.2% for the testis, caput, corpus, and cauda epididymis, respectively; *p* < 0.001), as well as type A (14.0% ± 5.2%, 26.6% ± 4.8%, 30.0% ± 8.7%, and 22.3% ± 3.1% for the testis, caput, corpus, and cauda epididymis, respectively; *p* < 0.05 for the testis, with no significant differences within the epididymis) and type P (3.0% ± 1.0%, 4.3% ± 1.8%, 9.3% ± 2.7%, and 10.0% ± 4.1% for the testis, caput, corpus, and cauda epididymis, respectively; *p* < 0.05, when the testis and *caput* are compared with the corpus and cauda, respectively).

## 3. Discussion

Melatonin and melatonin receptors are widely distributed in mammalian tissues and cells, including those of the reproductive system. In the female, granulosa cells surrounding the oocyte can synthesize melatonin [42], and the melatonin receptors have been found in the ovary [43], uterus [13,44], and mammary gland [14]. In the male, melatonin is secreted in the testes [34,45], and although there are several reports of the presence of melatonin receptors MT_1_ and/or MT_2_ in various organs of the male reproductive tract, such as the testes [16,17], epididymis [18], or prostate [19], to the best of our knowledge this is the first time that the presence of melatonin receptors MT_1_ and MT_2_ have been studied in the whole male reproductive tract of one species, the sheep.

Our study revealed that both melatonin receptors MT_1_ and MT_2_ are present in all of the organs of the ram reproductive tract. However, real-time PCR analyses showed a significantly higher genic expression of both receptors in the testis, the ampulla of the vas deferens, the seminal vesicle, and vas deferens.

For the MT_1_ melatonin receptor in the testis, Western blot analyses detected a strong 39 kDa band, compatible with the MT_1_ molecular weight [46], and a smaller band of 32 kDa that could be related to the receptor activation [7] along weaker bands of various molecular weights. These bands may be as a result of the oligomerization of the MT_1_ melatonin receptor, which can form dimers with itself, with MT_2_ receptors [47], or with other G protein–coupled receptors [48]. IHC also revealed that MT_1_ receptors were present in spermatogonias, spermatocytes, and spermatids. This multiple cellular location for MT_1_ could also explain the presence of those multiple bands detected in the Western blot, as the dimers formed could differ in the diverse cell types. As shown by IHC, the MT_2_ location in the testis is restricted to the neck of the newly-formed spermatozoa, which could also explain the low signal detected by the Western blot analysis, as the concentration of the MT_2_ receptor protein would be low relative to the total of the extracted testicular proteins. Our previous studies have also revealed the presence of the melatonin-synthesizing enzymes, aralkylamine N-acetyltransferase (AANAT) and N-Acetylserotonin O-methyltransferase (ASMT), in spermatocytes and spermatids in ram testes [34]. The presence of both melatonin synthesizing-enzymes and melatonin receptors in the same organ suggests that the testicular melatonin could act as an autocrine or paracrine molecule [49] and the locally-produced melatonin could protect the developing spermatozoa from free radical-mediated damage [50], which could impair ram fertility [51]. Our IHC analyses also revealed the absence of melatonin receptors in the Sertoli and Leydig cells of the ram testes. This result differs from those found in other species. The melatonin receptor MT_1_ has been previously detected in isolated Leydig cells from Syrian hamster testes [17], whereas both MT_1_ and MT_2_ receptors have been evidenced in isolated bovine Sertoli cells [52]. However, these differences could be due to the dissimilar identification methods for melatonin receptors.

The Western blot analyses revealed a high protein expression of both melatonin receptors in the epididymis, especially in the cauda epididymis, despite the low genic expression of these receptors detected by q-PCR. The Western blot analyses also revealed differences in the band pattern between the testis and caput, corpus, and cauda epididymis, and showed that the pattern observed in the cauda epididymis was more similar to the positive control (ejaculated spermatozoa) than those found in other portions of the epididymis or even in the testis. Moreover, the IHC analyses for MT_2_ revealed that the melatonin receptors were not located in the cauda epididymis tissue, but in the spermatozoa contained in its lumen.

In light of these results, we then investigated the location of both melatonin receptors on the sperm membrane during sperm maturation. Our findings revealed the presence of a new immunotype for MT_2_, (type N, with staining only in the neck), mainly confined to the testis, caput, and corpus epididymis. This melatonin receptor location has not been previously described in ejaculated ram spermatozoa [30,32]. However, this immunotype is the only one detected in ejaculated boar, bull, and red deer spermatozoa [53]. Our study also revealed that many of the newly-formed spermatozoa do not show melatonin receptors in their membranes when they are located in the testes, while both melatonin receptors are present when the spermatozoa reach the corpus epididymis. Furthermore, the percentage of the different immunotypes varied with the sperm passage through the epididymis. Therefore, when they reach the cauda, the immunotypes found are very similar to those found in the ejaculated semen [32]. These changes in the location of the melatonin receptors during epididymal transit might be related with the sperm maturation and the acquisition of fertilizing ability. During epididymal maturation, the sperm membrane undergoes changes in lipid and protein composition [54,55]. These changes may explain the variations we found in both the percentages of the melatonin receptor immunotypes and the Western blot band patterns between testis, caput, corpus, and cauda epididymis. In the head of the epididymis, a large part of testicular sperm surface protein, which could be masking these receptors, was lost [56]. This would explain the increase in the sperm rate showing melatonin receptors from the testis to the corpus epididymis revealed by the IIF technique. Moreover, it is known that melatonin receptors, and mainly MT_2_, can be desensitized by internalization [57], which could also explain the differences in melatonin receptor distribution observed during epididymal sperm transit, along with sperm heterogeneity.

On the other hand, both the ductus deferens and its secretory portion, the ampulla, showed high levels of m-RNA for both melatonin receptors. Both methods, the Western blot analyses and IHC staining, confirmed the presence of the receptors in these ram tissues. The presence of melatonin receptors in the vas deferens had been hypothesized by the use of 2-[^125^I]iodomelatonin in rats [20], and the results of the present study confirm the presence of both MT_1_ and MT_2_ receptors in the ram vas deferens. In this organ, the MT_1_ and MT_2_ receptors are located at the nucleus of the pseudostratified columnar epithelium cells, whereas MT_2_ is also located on the cytoplasm of the epithelial cells near the lumen, so its presence could be related to the secretory capacities of the vas deferens [58]. Until now, there are no reports on the presence and the possible effect of melatonin on the ampulla. In this work, we have located the MT_1_ melatonin receptor in the apical border of the epithelial cells of this organ, whereas the MT_2_ receptor is located in all the cytoplasm of the epithelial cells. The apical location of melatonin receptors, in contact with the ampulla lumen, suggests that these receptors might be stimulated by the melatonin secreted by the testis [34], which can reach this organ along with the spermatozoa and testicular and epididymal fluids. However, the possible effect of the pineal or testicular melatonin on the ampulla secretory activity needs to be assessed.

Finally, regarding the seminal vesicles, prostate, and bulbourethral glands, only the seminal vesicles showed high levels of gene expression of both melatonin receptors. These results differ from previous reports on the presence of melatonin receptors in human and rat prostate [19,59]. However, only the disseminated part of the prostate is present in the ram. This species lacks the prostate body where melatonin receptors are located in both rats and humans. In the seminal vesicles, IHC showed a nuclear location for the MT_1_ melatonin receptor, while that for MT_2_ was found in the cytoplasm. Very recent studies have demonstrated that melatonin treatment during the non-reproductive season reactivates the ram seminal vesicles [39] and increases their size in the Iberian ibex [40]. Therefore, it is likely that both effects may be exerted through the melatonin receptors we have here identified. Moreover, the seminal vesicle secretes a significant part of the seminal fluid that becomes semen. The increased volumes of ejaculate detected in ram during the reproductive season [24,60] or after exogenous melatonin treatment [26,61] could, therefore, be the result of pineal or exogenous melatonin, acting on the seminal vesicles through its receptors.

## 4. Materials and Methods

### 4.1. RNA Isolation and Retrotranscription

Tissue samples from the testis, epididymis (head, or caput; body, or corpus; and tail, or cauda), ampulla, prostate, seminal vesicle bulbourethral glands, and vas deferens, were collected from two Rasa Aragonesa rams during the reproductive season, in accordance with the directive 2010/63/EU of the European Parliament and of the Council of 22 September 2010 on the protection of animals used for scientific purposes (published in the Official Journal of the European Union, 20.10.2010, L 276/33), and immediately frozen in liquid nitrogen. Total RNA was extracted by the guanidine thiocyanate/phenol extraction method [62] by homogenization in 1 mL of TRI reagent (Sigma-Aldrich, St. Louis, MO, USA) per 100 mg of tissue. Traces of DNA were removed by DNAse treatment using a TURBO DNA-free DNA removal kit (Ambion, Invitrogen, Thermo Fisher Scientific, Waltham, MA, USA). RNA concentration was measured in a NanoDrop ND-100 Spectrophotometer (Thermo Fisher Scientific), and the integrity of the RNA was also verified by agarose gel electrophoresis.

To obtain the c-DNA, 500 ng of total RNA from each tissue and ram were reverse-transcribed using poly d(T)20 primers and the SuperScript First-Strand Synthesis System kit (Invitrogen, Thermo Fisher Scientific), following the manufacturer’s instructions.

To verify the retrotranscription, PCR amplification of glyceraldehyde-3-phosphate dehydrogenase (*GAPDH)* was carried out on the reverse-transcribed c-DNA from the ram reproductive tract tissues. A PCR mix composed by 2.5 µL of REDTaq DNA polymerase 0.05 U/µL (Sigma-Aldrich), 5 µL of 10× REDTaq DNA polymerase buffer (100 mM Tris-HCl pH 8.3, 500 mM KCl, 11 mM MgCl_2_, and 0.1% gelatin), 0.2 mM of each dNTP, (Invitrogen, Thermo Fisher Scientific) and 320 nM *GAPDH* primers, designed using the primer-BLAST tool and checked by BLAST analysis (NCBI, National Center for Biotechnology Information, U.S. National Library of Medicine, Bethesda, MD, USA) to verify gene specificity (Table 1) was added to 2 µL of the previously-obtained cDNA in a total volume of 50 mL. The cycling conditions consisted of 30 cycles of 30 s at 94 °C, 30 s at 58 °C, and 45 s at 72 °C. A 3 min denaturation step at 95 °C preceded cycling; at the end, a final 5 min extension at 72 °C was performed. PCR products were separated on 1% agarose gel in a Tris-borate-EDTA (TBE) buffer (Tris 0.9 M, boric acid 0.9 M and EDTA 20 mM, pH 8) containing 0.5 µL/mL ethidium bromide (Sigma-Aldrich) and were visualized under ultraviolet (UV) light. Molecular size was estimated by using 50 bp Dye Plus DNA Ladder (Takara Korea Biomedical Inc., Seoul, South Korea). Each PCR assay included a water control to rule out template contamination of PCR reagents.

### 4.2. Quantitative Real-Time PCR (qPCR)

The primers for the melatonin receptors MT_1_ and MT_2_ for qPCR (Table 1), along with the primers for housekeeping genes (*β-actin* and *GAPDH*), were designed using the primer-BLAST tool (NCBI, National Center for Biotechnology Information, U.S. National Library of Medicine, Bethesda, MD, USA), and Beacon Designer Free Edition software (Premier Biosoft International, Palo Alto, CA, USA) to rule out primer-dimer formation. Primer specificity was checked by BLAST analysis (NCBI, National Center for Biotechnology Information, U.S. National Library of Medicine, Bethesda, MD, USA).

To perform the real time PCR reaction, 2 µL of cDNA was used in a final volume of 20 µL containing 1× iTaq Universal SYBR Green supermix (Bio-Rad Laboratories, Inc., Hercules, CA, USA) and 200 nM for the *MT_1_* and *MT_2_* primers, or 300 nM for the *β-actin* and *GAPDH* primers. Real-time PCR reactions were performed in a StepOnePlus Real-Time PCR System (Applied Biosystems, Foster City, CA, USA). 40 PCR cycles were carried out. The cycling conditions consisted of a denaturation step of 15 s at 95 °C and an annealing/extension and plate read step at 60 °C for 1 min. A 2 min polymerase activation and DNA denaturation step at 95 °C preceded cycling. At the end, a melt-curve analysis was performed from 65 to 95°C, with 0.5 °C increments at 5 s/step. The ΔΔ*C*t method [63] was used to determine the expression of MT_1_ and MT_2_ receptors relative to *β-actin* and *GAPDH*, used as housekeeping genes. Tissues from each ram were analyzed twice, and cDNA from each tissue were loaded in triplicate in each qPCRreaction.

c-DNA obtained from ovine granulosa cells was used as a MT_1_ receptor positive control [64] whereas ovine retina was used as a MT_2_ receptor positive control [65].

### 4.3. Sodium Dodecyl Sulfate-Polyacrylamide Gel Electrophoresis (SDS-PAGE) and Western Blotting

Proteins from the testis, epididymis and accessory gland of the ram reproductive tract were extracted by adding 3 mL of extraction buffer composed of 125 mM Tris-HCl, 4% sodium dodecyl sulfate (SDS), 10% β-mercaptoethanol, 20% glycerol, and 0.02% bromophenol blue to 300 mg of tissue, followed by mechanical homogenization. After incubation at 100 °C in a sand bath for 4 min, the mix was centrifuged 7500× *g* for 5 min at 4 °C. The supernatant was recovered and, after adding 10% of a protease inhibitor cocktail (Sigma-Aldrich), was stored at −20 °C.

Proteins (10 µg) were loaded on 10% or 12% (*w*/*v*) acrylamide SDS-PAGE gels for the MT_1_ and the MT_2_ receptors, respectively. Proteins were separated by standard SDS-PAGE and transferred onto a PVDF membrane (Trans-Blot pack, Bio-Rad) using a transfer unit (Mini Trans Blot Electrophoretic Transfer Cell Unit, Bio-Rad). After the blocking of non-specific sites with 5% bovine serum albumin (BSA) in phosphate-buffered saline (PBS, 137 mM NaCl, 2.7 mM KCl, 8.1 mM Na_2_HPO_4_, and 1.76 KH_2_PO_4_, pH 7.2) for 4 h, the proteins were detected by incubating overnight at 4 °C with a rabbit primary antibody against the MT_1_ (GeneTex Inc, Irvine, CA, USA; Cat# GTX100003, RRID:AB_1241048) or the MT_2_ receptor (Acris Antibodies GmbH, Herford, Germany; Cat# AP01322PU-N, RRID:AB_1619198) diluted 1/1000 in 0.1% Tween-20 PBS containing 1% BSA. After extensive washing, this was followed by incubation for 1 h and 15 min at room temperature with a secondary donkey anti-rabbit IRDye 800-CW conjugated antibody (LI-COR Biosciences Lincoln, NE, USA; Cat# 926-32213, RRID:AB_621848) diluted 1:30,000. Finally, after washing, the membranes were scanned with the Odyssey Clx Infrared Imaging System (LI-COR Biosciences, Lincoln, NE, USA).

### 4.4. Immunohistochemistry

Tissue samples were fixed in Bouin solution for 90 min, dehydrated in a graded series of ethanol and embedded in paraffin. Sections (7 µm thick) were cut on a Surgipath microtome. The sections were exposed to immunohistochemical staining by an avidin-biotin complex technique (Vector Laboratories, Burlingame, CA, USA). Endogenous peroxidase was inactivated with 1.7% hydrogen peroxide in 100% ethanol for 30 min. The sections were then washed in PBS pH 7.4 and incubated in normal horse serum as a blocking reagent for 40 min, followed by overnight incubation at 4 °C with the specific primary antibody (rabbit polyclonal antibody against the MT_1_ receptor (RRID:AB_1241048, GeneTex, Irvine, CA, USA) and rabbit polyclonal antibody against the MT_2_ receptor (RRID:AB_1619198, Acris Antibodies GmbH, Herford, Germany), both at a dilution of 1:50 in PBS. Subsequently, the slides were incubated with biotinylated anti-rabbit antiserum (Vector Laboratories) for 30 min. An avidin-biotin-peroxidase complex (Vector Laboratories) was then applied for 40 min. The binding sites of the primary antibodies were visualized by diaminobenzidine (DAB) and peroxidase solution (0.12 g DAB in 240 mL of PBS pH 7.4 containing 3% H_2_O_2_) for 7 min. The slides were stained with Carazzi hematoxylin and mounted with 1,3-diethyl-8-phenylxanthine (DTX). As a negative control, samples were incubated with normal horse serum instead of the primary antibody, with the remaining procedure being the same.

### 4.5. Indirect Immunofluorescence Assays

The immature spermatozoa were obtained by mechanical homogenization from the testis and the caput, corpus, and cauda epididymis of five Rasa Aragonesa mature rams sacrificed in the slaughterhouse during the reproductive season.

The cells were fixed with 3.7% formaldehyde (*v*/*v*) in PBS for 20 min at room temperature. They were then centrifuged at 900× *g* for 5 min, and the pellet resuspended in PBS. After fixation, 40 µL of cell suspension was smeared onto poly-l-lysine-coated slides, and maintained at room temperature for 3 h to ensure good adhesion to the slide.

Samples were washed three times with PBS, and non-specific binding sites were blocked with 5% BSA (*w*/*v*) in PBS for 4 h at room temperature in a humidity chamber. After three washes in PBS, the spermatozoa were incubated overnight at 4 °C with the primary antibody (MT_1_ mouse polyclonal antibody (Abnova Corporation, Taipei City, Taiwan; Cat# H00004543-A01, RRID: AB_462681) for the melatonin receptor MT_1_ and rabbit polyclonal antibody for the melatonin receptor MT_2_ (RRID: AB_1619198, from Acris Antibodies GmbH, Herford, Germany), diluted 1 : 50 (*v*/*v*) in PBS containing 1% BSA (*w*/*v*). After three washes in PBS, the cells were incubated with the secondary antibody Alexa Fluor 594 chicken anti-mouse (Thermo Fisher Scientific; Cat# A-21201, RRID:AB_2535787) for the melatonin receptor MT_1_, and Alexa Fluor 488 chicken anti-rabbit (Thermo Fisher Scientific, Cat# A-21441, RRID:AB_2535859) for the melatonin receptor MT_2_, both diluted 1:800 (*v*/*v*) in PBS containing 1% BSA *w*/*v* for 1.5 h at room temperature in a humidity chamber and in the dark. The slides were then washed three times with PBS before the addition of 5 µL of 0.22 M triethylenediamine (DABCO) in glycerol:PBS (9:1 *v*/*v*) to enhance and preserve cell fluorescence. Finally, the preparations were covered with coverslips, sealed with colorless enamel and visualized using a Nikon Eclipse E-400 microscope (Kanagawa, Japan) under epifluorescent illumination. All samples were processed in duplicate in a blinded manner and at least 150 spermatozoa were scored per slide.

### 4.6. Statistical Analyses

The gene expression of MT_1_ and MT_2_ in ram reproductive tissues was evaluated by the Kolmogorov-Smirnov test to assess normal distribution. Differences between data were then analyzed by means of the Kruskal-Wallis test, followed by the Dunn’s multiple comparison test. In the gene expression analyses, granulosa cells and retina data was only used for positive control and representative purposes, and was not included in the statistical analyses.

The differences in the distribution of the melatonin receptors during epididymal maturation detected by IIF were analyzed by means of the Chi-square test.

All the statistical analysis was performed with SPSS software (v.15.0, IBM Software, Armonk, NY, USA).

## 5. Conclusions

In conclusion, in this study we have demonstrated the presence of the melatonin receptors MT_1_ and MT_2_ in all organs of the ram reproductive tract, with greater abundance in the testis, ampulla, seminal vesicles, and ductus deferens, although their functionality remains unclear and it needs to be further studied. We have also revealed that the location of these receptors in ram spermatozoa varies during their maturation and transit through the epididymis. These results open up new, interesting perspectives for further studies on the molecular mechanism of melatonin and the biological significance of its receptors.

## Figures and Tables

**Table 1:** Sequences of the *MT_1_*, *MT_2_*, *β-actin*, and glyceraldehyde-3-phosphate dehydrogenase *GAPDH* primers used in the real-time polymerase chain reaction (qPCR) study.

Gene	Accession Number	Forward Primer (5’-sequence-3’) (Position)	Reverse Primer (5’-sequence-3’) (Position)
*MT_1_*	NM_001009725.1	CTCCATCCTCATCTTCACCATC (164–185)	GGCTCACCACAAACACATTC (276–257)
*MT_2_*	NM_001130938.1	GCTGAGAGAATGGAGCGATATG (1692–1713)	GTCCACAGTGAGAAGCCATC (1772–1753)
*β-actin*	NM_001009784	CTCTTCCAGCCTTCCTTCCT (867–886)	GGGCAGTGATCTCTTTCTGC (1044–1025)
*GAPDH*	NM_001190390	CAAGGTCATCCATGACCACTTTG (516–538)	GTCCACCACCCTGTTGCTGTAG (1011–990)
